# Study on the Effect of Deep Eutectic Solvent Liquid Phase Microextraction on Quality Standard, Antitussive, and Expectorant of Sangbaipi Decoction

**DOI:** 10.1155/2021/9999406

**Published:** 2021-08-02

**Authors:** Lijing Li, Yuejie Wang, Fangxin Liu, Yang Xu, Huiwei Bao

**Affiliations:** ^1^College of Pharmacy, Changchun University of Chinese Medicine, Changchun 130117, China; ^2^College of Pharmacy, Baicheng Medical College, Baicheng 137000, China

## Abstract

The SD was extracted with a new green eutectic solvent, and the extraction method of TCM decoction was developed. In the quantitative analysis by HPLC, choline chloride phenol was selected as the eutectic solvent, THF was used as the extractant, and investigation of DES type, DES molar ratio, DES-to-THF ratio, vortex time, and material-to-liquid ratio was carried out. The experimental results showed that the optimal extraction method was as follows: the molar ratio of DES was 1 : 3, and the material-liquid ratio was 5 : 1200 (mL/*μ*L). The volume ratio of DES to THF was 1200 : 800 (*μ*L), the vortex time was 3 min, and the extraction was repeated two times. The eutectic solvent liquid phase microextraction method was adopted to optimize the extraction method of SD and reduce the complicated processing, long time, and low efficiency of traditional methods. At the same time, in the mouse ammonia water inducing cough and phenol red excretion and expectorant experiments, SD high- and medium-dose groups have a significant inhibitory effect on the frequency of antitussive in mice and both can increase the excretion of phenol red to varying degrees, indicating that SD has good cough-relieving and expectorant effect. The present study suggests a scientific basis and basis for the clinical research and quality standard formulation of SD.

## 1. Introduction

Sangbaipi decoction (SD), which is derived from Jingyue Quanshu written by Zhang Jingyue in the Ming Dynasty and composed of *Cortex Mori*, *Pinellia Ternata*, *Perilla*, *Apricot Kernel*, *Fritillariae Cirrhosae Bulbus*, *Gardenia Jasminoides*, *Scutellariae Radix,* and *Coptidis Rhizoma* [1], has the effect of clearing the lung and reducing Qi, resolving phlegm and stopping cough. It is mainly used to treat the lung with redundant Qi as well as asthma caused by fire and phlegm. In the prescription, *Cortex Mori* is used as the principal drug to facilitate the flow of the lung Qi to relieve asthma, promote diuresis, and detumescence; it is supplemented by *Scutellariae Radix*, *Coptidis Rhizoma*, and *Gardenia Jasminoides,* which can clear away phlegm heat; *Fritillariae Cirrhosae Bulbus*, *Apricot Kernel*, *Pinellia Ternata,* and *Perilla* can relieve asthma, phlegm, and the heat of tri-jiao. In April 2018, Sangbaipi decoction, an outstanding representative of traditional Chinese medicine (TCM) prescriptions, was officially selected into the first batch of the 100 antique classic prescriptions list issued by the State Administration of TCM. There are more and more studies on quality standard because of its wide application in modern clinical practice and definite curative effect, such as chronic bronchitis, chronic obstructive pulmonary disease, and mycoplasma pneumonia [2]. However, due to the characteristics of the preparation form of the decoction, such as the complex treatment process of the test sample, the need for concentration, and solvent extraction, there are many problems when establishing the quality standard, which greatly reduce the extraction efficiency.

Deep eutectic solvent (DES), firstly reported by Abbott Andrew et al. in 2003 [3], is a hypoeutectic mixture composed of hydrogen bond acceptor and hydrogen bond donor of solid halide salt with a certain stoichiometric ratio. It usually consists of two or three components, of which the melting point is significantly lower than that of each component [4]. Due to the interaction of hydrogen bonds in DES, charge delocalization makes the mixture formed as a liquid system at room temperature, of which the melting point is lower than that of each component [5–10]. As a new green and efficient solvent, DES has advantages of a simple preparation process, low cost and toxicity, environmental protection, complete biodegradation, and biocompatibility [11–13]. It is also reported to be applied in electrochemistry, preparation of nanomaterials, catalytic reaction, separation process, and preparation of functional materials [14–16]. In recent years, DES has extensively been used for extracting bioactive components from natural plant materials. So far, studies on SD are mainly focused on clinical research, instead of its effective components, extraction methods, antitussive, and expectorant effects. In this study, a new liquid-phase microextraction method [17] was employed for the separation of SD through optimizing the sample processing method of SD; in addition, in vivo experiments were also conducted to validate the effect of SD on antitussive and expectorant, so as to provide the basis for its clinical rational drug use and quality standard research.

## 2. Materials and Methods

### 2.1. Instrument

A Huapu S3000 high-performance liquid chromatography system (including a four-element low-pressure stirring pump, automatic sampler, column box, 1100 diode array detector, and chemical workstation) was used for chromatographic analysis. A KQ-250 ultrasonic cleaning machine produced by Kunshan Ultrasonic Instrument Co., Ltd. was used. An AB135-S electronic balance was purchased from Mettler-Toledo International Ltd. An ATY224 type-1/10000 balance, Shimadzu, Japan, was used. The Ql-901 vortex instrument was purchased from Haimen City Qilin Bell Instrument Manufacturing Co., Ltd., the HH-S4 digital constant-temperature water bath was purchased from Jiangsu Jinyi Instrument Technology Co., Ltd., the TDL-60B centrifuge was purchased from Shanghai Anting Scientific Instrument Factory, and the YLS-8A multifunction cough-inducing and asthma-inducing instrument was purchased from Jinan Yiyan Technology Development Co., Ltd. and BioTek Instruments, Inc.

### 2.2. Materials

*Cortex Mori*, *Pinellia Ternata, Perilla, Apricot Kernel, Fritillariae Cirrhosae Bulbus, Gardenia Jasminoides, Scutellariae Radix,* and *Coptidis Rhizoma* were purchased from Jilin Pharmacy and authenticated by associate professor Xiao Jinglei of the Department of Traditional Chinese Medicine Identification, Changchun University of Chinese Medicine, under the guidance of the Chinese Pharmacopoeia 2015 edition. The sources of the materials of SD is shown in Table 1. Berberine hydrochloride (batch no: 110713–201613, content 86.8%), palmatine hydrochloride (batch no: 110732–201611, content 86.8%), and baicalein (batch no: 111595–201607, content 97.9%) were purchased from the China Pharmaceutical Biological Products Analysis Institute (Beijing, China). Wogonin (batch no: asb-00023510-025, HPLC ≥ 98%) was obtained from Chromadex, USA. Methanol was added to an appropriate amount of the reference substance of palmatine hydrochloride, berberine hydrochloride, baicalein, and wogonin to prepare the mixed reference substance original solution with the concentration of 78, 108.6, 84.4, and 50.04 *μ*g/Ml, respectively, for the linear relationship investigation experiment; 1 mL of the mixed reference substance original solution precisely absorbed with methanol was diluted 10 times to obtain the mixed reference substance solution for HPLC analysis experiment.

Ammonia (Beijing Chemical Works, 20170516), phenol red (Tianjin Guangfu Fine Chemical Research Institute, 20110827), Chuanbei Loquat Ointment (Kyoto nianci'an Co., Ltd., g22003024), sodium bicarbonate (Tianjin Xintong Fine Chemical Co., Ltd., 20160506), and physiological saline (Jilin Dupang Pharmaceutical Co., Ltd., 1805290514) were used.

Methanol (batch no. 20200506) and acetonitrile (batch no. 20200501) in HPLC analysis were chromatographic grade and purchased from Fisher Company of the United States. Ultrapure water was purchased from Hangzhou Wahaha Co., Ltd. Tetrahydrofuran (lot no. 2019050801, Chengdu Cologne Chemicals Co., Ltd.) was used as solvent in microextraction and HPLC analysis. Phosphoric acid (batch no. 20170408) was purchased from Tianjin Guangfu Technology Development Co., Ltd. The source of DES is shown in Table 2.

SPF ICR mice were provided by Jilin Yisi experimental animal Co., Ltd., production license no. SCXK (Ji)—2016–0003. The abovementioned experimental animals were used by Changchun University of Chinese Medicine with the license number of SCXK (Ji)—2016–0017. The ethics number of laboratory animal was 201908A019. They were reared in the experimental animal center of Changchun University of Chinese Medicine and divided into male and female cages, with rat pellet feed and free drink. The room temperature was 20–23°C, and the humidity was 44%–57%. The pellet feed of SPF-grade laboratory mice was provided by Jilin Yisi Experimental Animal Co., Ltd.

### 2.3. Chromatographic Conditions

Alltima^TM^ C_18_ column: 250 mm × 4.6 mm, 5 *μ*m, column temperature: 30°C, detection wavelength: 238 nm, injection volume: 20 *μ*L, flow rate: 1 mL/min, mobile phase: acetonitrile (A)-0.1% phosphoric acid aqueous solution (B), and gradient elution: 0–18 min, 28% A; 18–20 min, 28 ⟶ 50%; 20–26 min, 50% A; 26–28 min, 50 ⟶ 60% A; and 28–35 min, 60% A.

### 2.4. Preparation of SD

According to the preparation method of SD in Jingyue Quanshu, 2.4 g of *Cortex Mori, Pinellia Ternata, Perilla, Apricot Kernel, Fritillariae Cirrhosae Bulbus, Gardenia Jasminoides, Scutellariae Radix,* and *Coptidis Rhizoma* was weighed and soaked in 400 mL distilled water and decocted for 320 mL, and the filtrate was filtered and reserved.

### 2.5. Synthesis of DES

The hydrogen bond donor and hydrogen bond acceptor were mixed according to the molar ratio, which was heated in a water bath at 80°C∼100°C, and fully stirred in a beaker until a clear, transparent, and slightly viscous liquid solvent was obtained for preparing DES.

### 2.6. Deep Eutectic Liquid-Phase Microextraction Process (DES-LPME)

**Figure 1:**
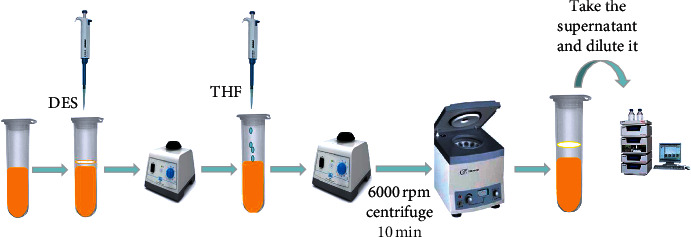
Deep eutectic liquid-phase microextraction process.

The process of DES-LPME is shown in Figure 1. 1200 *μ*L DES was added into the test tube with 5 mL SD and then vortexed for 5 min to premix. Then, 800 *μ*L tetrahydrofuran (THF) was added to the test tube for mixing and centrifuged at 6000 rpm for 10 min. The DES phase was separated from the other phase. The upper phase (DES) was removed from the test tube with a pipette gun to obtain the extract. The step was required to repeat two times. The first and second extractions were removed and placed in a 5 mL volumetric flask with THF at constant volume and mixed evenly, and 0.2 mL, acetonitrile to 5 mL, was taken for HPLC determination.

### 2.7. Study on Antitussive and Antitussive Effects of SD on Mice

The mice were randomly divided into a model group, Chuanbei Loquat Ointment group (4.55 mL/kg), SD with low-dose group (12.125 mL/kg), SD with medium-dose group (24.25 mL/kg), and SD with high-dose group (48.5 mL/kg), with 10 mice in each group, male and female in half. The mice in experiment groups were administrated by required dose, compared to the model group given the same dose of distilled water by gavage for 5 days. After the last administration of 30 min in the antitussive experiments, the mice were placed in the ammonia water coughing device, which is the YLS-8A multifunction cough-inducing asthma apparatus, as well as the 50% ammonia constant-pressure spray for 30 s. The cough was recorded at the first time in the mice (mice largely opened mouth, accompanied by abdominal contraction and occasional cough), which was cough latency, as well as the cough frequency within 3 min at the same time. After the last administration of 30 min in the phlegm experiment, 0.5% phenol red solution (0.2 mL/10 g) was injected intraperitoneally. After 30 minutes, the rats were killed, and the neck was cut open to expose the trachea. A section of the trachea from the thyroid gland to the trachea branch was cut off and put into the mixture of 2 mL sodium bicarbonate and normal saline (the ratio was 1 : 15). The absorbance (OD value) at 546 nm was detected by using an enzyme reader for calculating the phenol red excretion.

## 3. Results and Discussion

### 3.1. Evaluation of the Method

Under optimal conditions, system suitability testing, linear range (LR), correlation coefficients (R), limit of detection (LOD), limit of quantification (LOQ), accuracy test and relative standard deviation (RSD), and rate of recovery were investigated to evaluate the proposed HPLC-DAD method.

**Figure 2:**
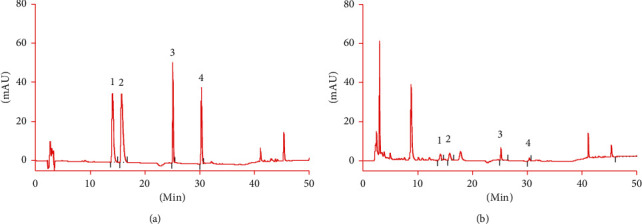
HPLC chromatogram: (a) mixed reference substance; (b) test substance; 1- palmatine; 2- berberine; 3- baicalein; and 4- wogonin.

The system adaptability test showed that the chromatographic peaks of palmatine, berberine, baicalein, and wogonin were well separated, with more than 3000 theoretical plates. The results are shown in Figure 2.

The standard calibration curves were constructed using a series of blank samples spiked with the standard substance at different concentrations. The analytical results (Table 3) show that there was a good linear relationship with the peak area in the respective concentration range.

The LOD was calculated based on a signal-to-noise ratio of 3, while LOQ was obtained based on the signal-to-noise ratio of 10. The results are shown in Table 3.

The precision was tested by intraday (*n* = 3) and interday (*n* = 3) analysis, and the RSDs were within 1.13–1.76% and 1.07–1.88%, respectively.

Six SDs (5 mL each) were extracted according to the “2.6” extraction process. HPLC analysis showed that the contents of palmatine, berberine, baicalein, and wogonin were 101.85 *μ*g/mL, 262.51 *μ*g/mL, 13.28 *μ*g/mL, and 12.06 *μ*g/mL, respectively, with RSD values of 2.25%, 1.97%, 1.81%, and 1.48%.

The stable test of the extraction of six SDs and the RSD values of palmatine, berberine, baicalein, and wogonin were 0.64%, 0.52%, 1.48%, and 1.25%, respectively, which indicated that the extract was stable within 24 hours.

To determine the accuracy of the developed method, 0.5 mL of palmatine reference solution (251 *μ*g/mL), 1 mL of berberine reference solution (641 *μ*g/mL), and baicalein reference solution were added to the selected samples of SDs (35.7 *μ*g/mL) 0.5 mL and wogonin reference substance solution (30.55 *μ*g/mL) 0.5 mL. After extraction and analysis, the average recovery rate of palmatine, berberine, baicalein, and wogonin is calculated to be in the range of 97.51–98.45%, and the RSD value is in the range of 1.34–1.49%.

By changing the composition of the mobile phase, including methanol-phosphoric acid-water, acetonitrile-phosphoric acid-water, methanol-water, and acetonitrile-water, we found that the acetonitrile-phosphoric acid-water system can separate the sample well to achieve good resolution, with good chromatographic peak shape of the components to be determined. Using the HPLC-DAD method to scan the full wavelength of the sample, it is found that the peak shape is at 238 nm, the resolution is better, the number of peaks is higher, and the peak response value is higher. Therefore, 238 nm was selected as the detection wavelength of mulberry SD. Thus, based on good stability, precision, and repeatability, the method could be used for the accurate and rapid determination of palmatine, berberine, baicalein, and wogonin in SD.

### 3.2. Screening on DES Species

The hydrogen bond donor and acceptor were mixed according to the molar ratio. The mixture was heated in a water bath at 80–100°C and stirred well in a beaker until a clear, transparent, and slightly viscous liquid solvent was obtained for preparing DES.

The hydrogen bond donor and receptor shown in Table 4 were mixed in a certain molar ratio, and DES was prepared by the “2.5” method and extracted according to the “2.6” experimental steps.

The results showed that the two phases of DES formed by choline chloride and phenol could be completely separated by THF when extracting with THF, whereas the other eutectic solvents could not be separated by THF. Therefore, THF as an extractant and choline chloride phenol as a eutectic solvent were selected for subsequent experiments.

Due to the hydrophobic nature of DES and THF, as well as good solubility to palmatine, berberine, baicalein, and wogonin, the combination is suitable for extracting some active components from an aqueous solution and aqueous suspension for pretreatment on concentration or determination of active components in the TCM decoction.

### 3.3. Extraction Times

The times of extraction is the key factor affecting the whole extraction process. Choosing the appropriate times of extraction can not only improve the extraction effect but also save the experimental cost and shorten the experimental time.

According to the extraction rate of palmatine, berberine, baicalein, and wogonin in the solution since each extraction, we had found that the extraction rate of palmatine, berberine, baicalein, and wogonin in SD could be significantly improved by using choline chloride phenol as eutectic solvent and THF as extractant. Wogonin could be completely extracted after the first extraction; moreover, the extraction rate of palmatine, berberine, and baicalein can reach more than 93% after two extractions, so the solution with twice extractions was selected for the study.

### 3.4. Effect of DES Molar Ratio

**Figure 3:**
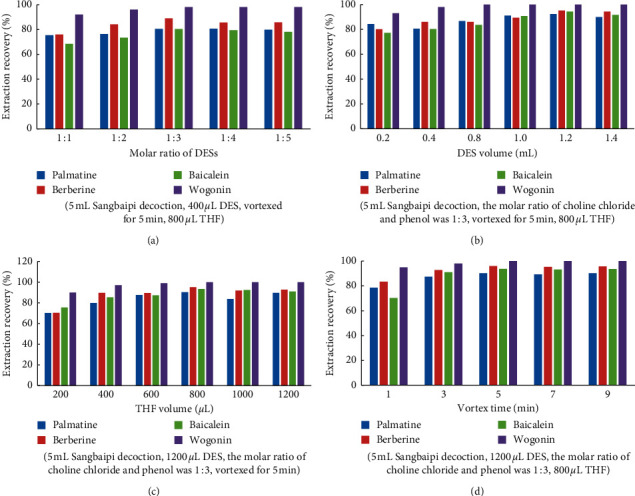
Effects of various factors on the extraction rate of palmatine, berberine, and baicalein in SD. (a) Effect of the molar ratio of DES, (b) effect of the DES volume, (c) effect of the THF volume, and (d) effect of the vortex time.

In the study, the selection of a suitable extraction solvent played an important role in the most effective separation process. We chose choline chloride and phenol as an effective separation solvent, which were made into five kinds of DES with different molar concentrations (1 : 1, 1 : 2, 1 : 3, 1 : 4, and 1 : 5). The results are shown in Figure 3(a). When the molar ratio of choline chloride to phenol was increased to 1 : 3, the extraction rate of palmatine, berberine, and baicalein was significantly increased, and then, the extraction rate had no significant change. So, the molar ratio of choline chloride and phenol was 1 : 3.

### 3.5. The Influence of the Proportion of Extraction

Optimizing the solid-liquid ratio is of great significance in the study of liquid-phase microextraction. Different volumes of eutectic reagents (5 : 0.4, 5 : 0.6, 5 : 0.8, 5 : 1.0, 5 : 1.2, and 5 : 1.4) were added to a 5 mL sample solution of SD. We selected the best ratio of material to liquid through the extraction effect. Results are as shown in Figure 3(b); the extraction rate of palmatine, berberine, and baicalein in SD was the highest when the DES dosage was 1200 *μ*L.

### 3.6. The Influence of the Ratio of DES to THF

As a solvent, tetrahydrofuran (THF) was used to remove DES from the aqueous phase. In this deep eutectic study, THF was used to separate and enrich the DES phase and aqueous phase. After adding THF, the DES phase and water phase of SD were separated efficiently and thoroughly. The effects of different volume ratios of DES and THF (1200 : 200, 1200 : 400, 1200 : 600, 1200 : 800, 1200 : 1000, and 1200 : 1200) on the extraction rate of SD were compared. The results are shown in Figure 3(c). When the volume ratio of DES to THF is 1200 : 800, the extraction rate of palmatine, berberine, and baicalein is the best.

### 3.7. The Influence of Vortex Time

In the study of liquid-phase extraction, the contact time between the sample solution and extract is particularly important. Our research investigated the effects of a vortex time of 1 min, 3 min, 5 min, 7 min, and 9 min on the extraction rate of palmatine, berberine, and baicalein in SD. The results are shown in Figure 3(d). The results showed that the extraction rate of palmatine, berberine, and baicalein reached the highest when the vortex time had lasted for 5 min. With the extension of vortex time, the extraction rate did not change significantly. Therefore, under the premise of high efficiency and accuracy, the vortex time of SD was 5 min.

### 3.8. Box–Behnken Response Surface Method Test

#### 3.8.1. Response Surface Design and Results

Combining the single-factor test, according to the Box–Behnken principle, the Design-expert 8.0.6 software is used to design a three-factor three-level test program. The test contains 5 central points and 17 test points, with palmatine and berberine. The content of baicalein is subjected to dimensionless treatment to obtain the comprehensive evaluation value as the inspection index, and the experiment is carried out. The response surface optimization conditions are shown in Table 5, and the design plan results are shown in Table 6.

#### 3.8.2. Quadratic Regression Model Fitting and Model Analysis

**Figure 4:**
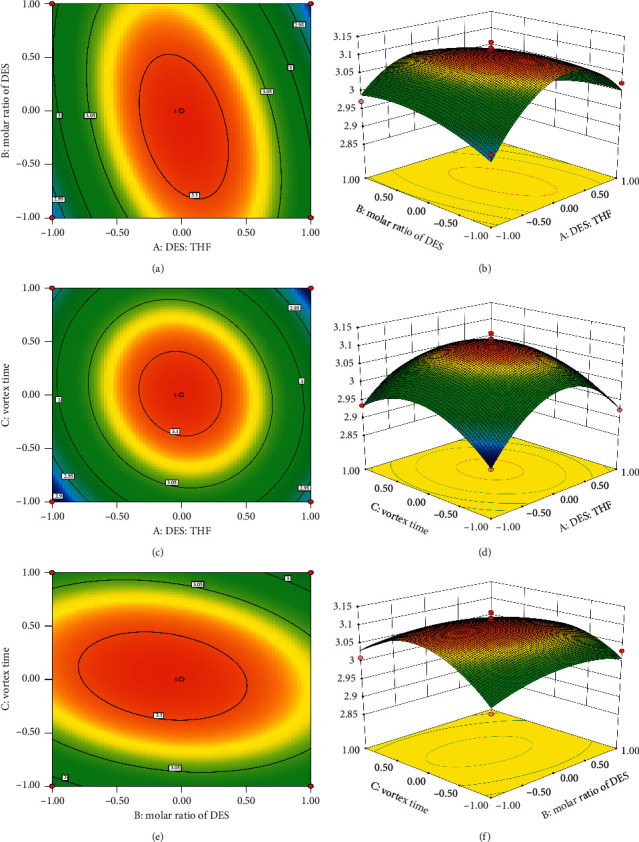
Response surface and contour of the interaction of various factors.

Using the response surface to perform regression fitting on the experimental data, the multinomial regression simulation equation of the extraction rate of SD is obtained as Y = 3.11 – 1.883*E* − 003*A* − 8.845*E* − 003*B* + 1.969*E*−003*C* − 0.043AB − 0.023AC − 0.022BC − 0.13*A*^2^ − 0.033*B*^2^ − 0.081*C*^2^. It can be seen from Table 7 that the high F value (23.10) and low *P* value (0.0002) of this test result indicate that the model has extremely significant differences. The *F* value of the lack-of-fit term is 2.79 and the *P* value is 0.1731, indicating that the lack of fit is not significant, that is, the model has a good fit. The *R*^2^_Adj_ of this model is 0.9256 > 0.80, indicating that this model can explain at least 92.56% of the response value change. *R*^2^ = 0.9674, indicating that the actual value has a good correlation with the predicted value of the equation. The signal-to-noise ratio Adeq Precision = 12.539 > 4 indicates that the model has a very high degree of fit and reliability. From the F value of each individual item, it can be seen that the degree of influence of the analysis conditions on the extraction rate of SD in descending order is *B* (molar ratio of DES), *C* (vortex time), and *A* (the ratio of DES to THF)). From the *F* value of the quadratic term, it can be seen that the difference between *A*^2^, *B*^2^, and *C*^2^ on the response value is extremely significant, the *F* value is generally higher, and in the regression equation, the coefficients of these three terms are all negative values. It shows that when the maximum value of this factor is exceeded, as the ratio of DES to THF, molar ratio of DES, and vortex time increase, the extraction rate will decrease. This conclusion is consistent with the single-factor test results. In this model, *A*^2^, *B*^2^, and *C*^2^ have extremely significant effects on the extraction rate of SD. It can be seen from Figure 4 that the contour map of the AB interaction is obviously elliptical, indicating that the AB interaction has a significant impact.

Optimized by Design-expert 8.0.6 software, the optimal extraction process conditions of SD were as follows: DES: THF was 1200 : 803.28 (v: v), the molar ratio was 1 : 2.84, and the vortex time was 3.12 min. Combining the actual operation situation, the final determination was that the extraction process had DES: THF 1200 : 800 (v: v), the molar ratio 1 : 3, and the vortex time 3 min.

#### 3.8.3. Verification Test

5 mL of SD was transferred, and 3 verification experiments were performed according to the optimized extraction process. The average extraction rates of palmatine, berberine, and baicalein were 92.02%, 94.98%, and 92.67%, respectively. The comprehensive evaluation value was 3.138. The relative error with the predicted value was less than 2%. It was suggested that the optimized extraction process was stable and reliable.

### 3.9. Comparison in Determination Results of Samples

To verify the accuracy of the sample processing method, 10 SD samples were prepared by the deep eutectic solvent liquid-phase microextraction method, direct determination method, and freeze-drying extraction method. The deep eutectic solvent liquid phase microextraction method was adopted according to “2.6”; the direct determination method was the SD solution, filtered through a 0.22 *μ*m microporous membrane; the freeze-drying extraction method was to take 5 mL SD to freeze-dry till powder, perform ultrasonic extraction at 60°C for 30 min after adding 50 mL methanol, then filter and dry the obtained filtrate by vacuum, and fix the volume to 5 mL; in terms of the three methods, contents of palmatine, berberine, baicalein, and wogonin in SD were determined as shown in Table 8. According to the results in Table 9, by comparing the same SD determined by different treatment methods, we had found the results in depth, and accurateness of the eutectic solvent liquid-phase microextraction method and freeze-drying extraction method was higher and more than that of the direct determination method.

The TCM decoction contains a large amount of water, which diluted the content of active ingredients to a low level. It is often necessary to concentrate the decoction due to the difficulties in the determination of TCM decoction for making the concentration of the test sample above the limit of quantification and detection. The concentration of water is often accompanied by heating, which may destroy the content of active ingredients [18], as well as complex treatment. The freeze-drying method can also be used in the concentration of samples, which is also more complex, time consuming, and less efficient. In spite of high active ingredients in some decoctions, the determination method of direct filtration will lose some precipitates with poor solubility in water [19], which makes the determination result lower than the real result.

### 3.10. Study on Antitussive and Expectorant Effects of SD on Mice

#### 3.10.1. Antitussive Effect of SD on Mice

Cough, as a protective respiratory reflex action, is a common symptom of bronchial asthma [20], pneumonia, and other diseases. Ammonia water [21] is used as a chemical stimulator. When the body is attacked by this kind of chemical factor, it stimulates the respiratory receptor and then causes cough.

After induced by ammonia, the mice in each group had cough reaction at different times. Compared with the model group, the SD group with high, medium, and low doses, as well as the Chuanbei Loquat Ointment group, could significantly prolong the cough latency of mice (*P* < 0.05 or *P* < 0.001); the SD group with high and medium doses, as well as the Chuanbei Loquat Ointment group, could also significantly inhibit the cough frequency of mice (*P* < 0.05 or *P* < 0.001). Therefore, SD has a good antitussive effect. The results are shown in Table 8.

#### 3.10.2. Study on the Expectorant Effect of SD on Mice

Sputum is the secretion of the human respiratory tract, which is secreted by the trachea, bronchus, nose, and throat. Under the normal physiological state, the mucus secreted by the respiratory tract is very little, which can keep the respiratory tract moist. However, when inflammation occurs, the sputum will accumulate, is viscous, and is difficult to discharge. In the expectorant experiment, phenol red [22, 23] was injected intraperitoneally to reach the lung through blood circulation, which could be detected in the trachea wall under the secretion of the trachea.

Phenol red excretion and expectorant test in mice: First, the standard curve of phenol red solution was established. A series of phenol red solutions with the concentrations of 0, 0.1, 0.2, 0.4, 0.5, and 1.0 *μ*g/mL were prepared by taking an appropriate amount of phenol red and using 1 mol/L sodium hydroxide solution. The absorbance values were determined according to the instrument. The standard curve equation was *Y* = 0.0692*X* + 0.0471 (*r* = 0.9998) by linear regression with phenol red concentration (*X*, *μ*g/mL) as the abscissa and absorbance value (*Y*) as the ordinate.

Compared with the model group, the SD groups with the high, medium, and low doses and Chuanbei Loquat Ointment could increase the excretion of phenol red in different degrees, with a significant effect (*P* < 0.05 or *P* < 0.001) in SD groups with the high and medium doses and the Chuanbei Loquat Ointment group, which showed that SD improved the secretion of sputum in the trachea of mice and had an expectorant effect. The results are shown in Table 10.

## 4. Conclusions

This study provides a new method for the determination and extraction of active components in SD. According to deep eutectic solvent liquid-phase microextraction (LPME), we selected phenol and choline chloride as the deep eutectic solvent to extract and determine four active components, such as palmatine, berberine, baicalein, and wogonin, so as to establish the quality evaluation method of SD. Compared with other methods, this method has the advantages of simple operation, good repeatability, high accuracy, and efficiency, as well as low pollution. In the other way, it is proved that SD has good antitussive and expectorant effects through the experiments of ammonia-induced cough and phenol red excretion and expectorant in mice.

## Figures and Tables

**Table 1:** Sources of the materials of SD.

Materials	Source (province)	Materials	Source (province)
*Cortex Mori*	Henan	*Rhizoma Coptidis*	Sichuan
*Pinellia Ternata*	Henan	*Scutellaria Baicalensis*	Jilin
*Gardenia Jasminoides*	Henan	*Perilla Frutescens*	Hubei
Apricot kernel	Jilin	*Fritillaria Thunbergii*	Jilin

**Table 2:** Sources of deep eutectic solvents.

Reagent	Sources	Batch number
Choline chloride	Shanghai Zhanyun Chemical Co., Ltd.	20190120
Maltose	Zhengzhou Kangyuan Chemical Products Co., Ltd.	201901101
Malic acid	Zhengzhou Kangyuan Chemical Products Co., Ltd.	201904201
Fructose	Zhengzhou Kangyuan Chemical Products Co., Ltd.	202001104
Phenol	Xilong Science Co., Ltd.	20190401
Propylene glycol	Tianjin Zhonghe Shengtai Chemical Co., Ltd.	20180309
Urea	Tianjin Ruijinte Chemicals Co., Ltd.	20170109
Acetic acid	Tianjin Ruijinte Chemicals Co., Ltd.	20180402
Citrin	Zhengzhou Kangyuan Chemical Products Co., Ltd.	201904701

**Table 3:** Linear relationships of various constituents.

Ingredient	Regression equation	Linear range/ (*μ*g/mL)	*r*	LOD/ (*μ*g/mL)	LOQ/(*μ*g/mL)
Palmatine	*Y* = 28.072*X* + 52.459	0.39∼78	0.9996	0.11	0.36
Berberine	*Y* = 36.045*X* − 27.853	0.543∼108.6	0.9995	0.14	0.46
Baicalein	*Y* = 12.978*X* + 36.503	0.422∼84.4	0.9996	0.09	0.30
Baicalin	*Y* = 16.184*X* + 16.13	0.2502∼50.04	0.9997	0.11	0.36

**Table 4:** Screening on DES species.

No.	Abbreviation	Hydrogen bond receptor	Hydrogen bond donor	Mole ratio	Appearance
1	DES-1	Choline chloride	Maltose	1 : 1	Incomplete separation from THF
2	DES-2	Choline chloride	Malic acid	1 : 1	Incomplete separation from THF
3	DES-3	Choline chloride	Lactate	1 : 2	Incomplete separation from THF
4	DES-4	Choline chloride	Fructose	1 : 1	Incomplete separation from THF
5	DES-5	Choline chloride	Phenol	1 : 3	Completely separated from THF
6	DES-6	Choline chloride	Glycerol	1 : 2	Incomplete separation from THF
7	DES-7	Choline chloride	Propylene glycol	1 : 2	Incomplete separation from THF
8	DES-8	Choline chloride	Xylitol	1 : 1	Incomplete separation from THF
9	DES-9	Choline chloride	Urea	1 : 2	Incomplete separation from THF
10	DES-10	Choline chloride	Acetic acid	1 : 2	Incomplete separation from THF
11	DES-11	Choline chloride	Citric acid	1 : 2	Incomplete separation from THF

**Table 5:** Response surface optimization conditions.

Level	Factor		
	*A* (DES: THF, v: v)	*B* (molar ratio of DES)	C (vortex time, min)
−1	1200 : 600	1 : 2	3
0	1200 : 800	1 : 3	5
1	1200 : 1000	1 : 4	7

**Table 6:** Design plan results.

No.	*A*	*B*	*C*	Comprehensive index
1	−1	−1	0	2.941
2	1	0	1	2.885
3	0	0	0	3.113
4	0	0	0	3.120
5	0	0	0	3.135
6	−1	0	1	2.936
7	0	1	−1	3.030
8	0	0	0	3.085
9	1	−1	0	3.023
10	0	0	0	3.110
11	0	1	1	2.986
12	0	−1	−1	2.968
13	1	0	−1	2.923
14	0	−1	1	3.011
15	1	1	0	2.883
16	−1	1	0	2.972
17	−1	0	−1	2.880

**Table 7:** Variance analysis of the Box–Behnken test.

Variance source	Sum of squares	Degree of freedom	Mean square	*F* value	*P* value	Significance
Model	0.12	9	0.013	23.10	0.0002	Significant
*A*	2.835*E* − 005	1	2.835*E* − 005	0.050	0.8303	
*B*	6.258*E* − 004	1	6.258*E* − 004	1.09	0.3306	
*C*	3.103*E* − 005	1	3.103*E* − 005	0.054	0.8226	
AB	7.323*E* − 003	1	7.323*E* − 003	12.79	0.0090	
AC	2.189*E* − 003	1	2.189*E* − 003	3.82	0.0915	
BC	1.904*E* − 003	1	1.904*E* − 003	3.32	0.1111	
*A* ^2^	0.066	1	0.066	115.22	<0.0001	
*B* ^2^	4.495*E* − 003	1	4.495*E* − 003	7.85	0.0265	
*C* ^2^	0.028	1	0.028	48.81	0.0002	
Residual	4.009*E* − 003	7	5.727*E* − 004			
Lack of fit	2.714*E* − 003	3	9.047*E* − 004	2.79	0.1731	Not significant
Pure error	1.295*E* − 003	4	3.238*E* − 004			
Cor total	0.12	16				
*R* ^2^	0.9674					
*R* ^2^_Adj_	0.9256					

**Table 8:** Effect of SD on mice with cough induced by ammonia ($\bar{x}\pm s$, *n* = 10).

Group	Dose (mL/kg)	Cough latency period (s)	Cough frequency
Model group	—	36.7 ± 14.22	32.4 ± 5.72
Chuanbei loquat ointment group	4.55	54.0 ± 14.74^*∗*^	25.0 ± 6.41^*∗*^
SD group with high dose	48.5	61.8 ± 8.05^*∗∗*^	21.2 ± 5.57^*∗∗*^
SD group with medium dose	24.25	56.8 ± 4.69^*∗∗*^	24.0 ± 6.83^*∗*^
SD group with low dose	12.13	54.8 ± 10.81^*∗∗*^	31.7 ± 13.56

*Note.* Compared with the control group, ^*∗*^*p* < 0.05, ^*∗∗*^*p* < 0.001.

**Table 9:** Application of the novel method to SD samples (*N* = 10).

No.	Batch number	Palmatine	Berberine	Baicalein	Wogonin
		(*μ*g/mL)	(*μ*g/mL)	(*μ*g/mL)	(*μ*g/mL)
1	Deep eutectic solvent microextraction	102.56 ± 5.89	262.88 ± 19.43	14.23 ± 2.13	11.64 ± 1.52
2	Freeze-drying extraction	105.11 ± 7.18	267.41 ± 21.98	14.37 ± 2.62	11.59 ± 1.98
3	Direct determination method	83.13 ± 3.81^*∗*^	237.34 ± 13.13^*∗*^	11.31 ± 1.58^*∗*^	9.42 ± 1.65^*∗*^

*Note.* Compared with the DES group, ^*∗*^*p* < 0.01.

**Table 10:** Effect of SD on phenol red excretion in the trachea of mice ($\bar{x}\pm s$, *n* = 10).

Group	Dose (mL/kg)	Phenol red concentration (*μ*g/mL)
Model group	—	0.35 ± 0.10
Chuanbei Loquat Ointment Group	4.55	0.55 ± 0.23^*∗*^
SD group with high dose	48.5	0.61 ± 0.30^*∗*^
SD group with medium dose	24.25	0.54 ± 0.18^*∗∗*^
SD group with low dose	12.13	0.41 ± 0.19

*Note.* Compared with the control group, ^*∗*^*p* < 0.05, ^*∗*^*p* < 0.01.

## Data Availability

The main table and figure data used to support the findings of this study are included within the article.
